# Long term delivery of pulsed magnetic fields does not alter visual discrimination learning or dendritic spine density in the mouse CA1 pyramidal or dentate gyrus neurons

**DOI:** 10.12688/f1000research.2-180.v2

**Published:** 2013-12-04

**Authors:** Matthew Sykes, Kalina Makowiecki, Jennifer Rodger

**Affiliations:** 1 Experimental and Regenerative Neurosciences, School of Animal Biology, University of Western Australia, Crawley, Australia

## Abstract

Repetitive transcranial magnetic stimulation (rTMS) is thought to facilitate brain plasticity. However, few studies address anatomical changes following rTMS in relation to behaviour. We delivered 5 weeks of daily pulsed rTMS stimulation to adult ephrin-A2 ^-/-^ and wildtype (C57BI/6j) mice (n=10 per genotype) undergoing a visual learning task and analysed learning performance, as well as spine density, in the dentate gyrus molecular and CA1 pyramidal cell layers in Golgi-stained brain sections. We found that neither learning behaviour, nor hippocampal spine density was affected by long term rTMS. Our negative results highlight the lack of deleterious side effects in normal subjects and are consistent with previous studies suggesting that rTMS has a bigger effect on abnormal or injured brain substrates than on normal/control structures.

## Introduction

Repetitive transcranial stimulation (rTMS) generates electrical currents in the brain by electromagnetic induction and has been shown to induce synaptic plasticity in human and animal models ^1^. Importantly, rTMS induces long term potentiation (LTP) in rodent hippocampus *in vitro* ^2^ and several sessions of high-frequency rTMS increases the capacity to induce LTP compared to untreated controls, suggesting it may also regulate metaplasticity ^3, 4^. Because rTMS acts on the same plasticity mechanisms as learning and memory, it has been hypothesised that rTMS may serve as a "priming" mechanism to facilitate long-term synaptic and structural modifications ^5, 6^. The implication is that repeated rTMS stimulation sets up a "plastic" brain state that is conducive to long term functional and structural changes ^5^. For this reason, rTMS is being explored in combination with behavioural training tasks to see whether it can be used to prime or improve learning and cognitive performance in humans ^7, 8^. However, the potential mechanisms whereby rTMS might accelerate learning remain unknown.

Declarative and spatial learning tasks are strongly associated with the hippocampus. More specifically, hippocampal dendritic spines have been identified as the likely loci of activity-dependent synaptic plasticity and possible structural correlates of memory and learning ^9, 10^. High dendritic spine density in hippocampal neurons is associated with learning ability and higher performance on cognitive tasks ^11– 14^. Furthermore, LTP and LTD induce structural changes in dendritic spines, with LTP induced electrically or via learning increasing dendritic length and spine density ^15, 16^. Because higher spine density is associated with higher spine mobility and turnover rates ^15, 16^, this measure is thought to reflect a greater capacity for synaptic reorganisation.

To date, the only study to examine changes in dendritic spines after rTMS did so following a single stimulation and showed no change in spine density, although the size of the smallest spines was increased ^2^. Therefore, very little has been done to investigate the impact of long term rTMS on spine density in the hippocampus, or how it might interact with the learning process. Given the significant structural changes induced in the mouse visual system following repeated stimulation sessions, and evidence for structural changes in the human brain ^17^, we hypothesised that a similar long-term rTMS regime in combination with a hippocampus-dependent learning task, would rescue impaired learning strategies previously found in ephrin-A2 ^-/-^ mice ^18^ and alter spine density in the hippocampus.

We delivered 5 weeks of daily pulsed rTMS stimulation to ephrin-A2 ^-/-^ and wildtype mice undergoing a visual learning task and analysed learning performance, as well as spine density in the dentate gyrus molecular and CA1 pyramidal cell layers in Golgi-stained material.

We used ephrin-A2 ^-/-^ mice because they have previously been shown to have a specific learning deficit ^18^. In addition, although ephrin-A2 is expressed in the mouse hippocampus throughout life and has been implicated in its topographic organisation ^19, 20^, there is no evidence that it is involved in synaptic plasticity or spine dynamics ^21^. Thus we aimed to examine a learning-mediated effect of rTMS on dendritic spines. Although mice of both genotypes learned the task, their performance remained suboptimal due to lack of motivation to obtain food rewards through insufficient food restriction ^22^ and neither learning behaviour, nor hippocampal spine density were affected by long term rTMS. Our negative results are consistent with previous data showing that rTMS has a selective effect on abnormal or injured brain circuitry ^23^, and the lack of deleterious side effects observed in normal human subjects ^8, 24^.

## Methods

### Animals

This experiment used 10 wildtype (C57Bl/6J) and 10 ephrin-A2 ^-/-^ knockout mice, with equal number of males and females. Wildtype mice were purchased from Animal Research Centre (Canning Vale, WA, Australia). Ephrin-A2 ^-/-^ mice were a generous gift from David Feldheim ^25^ and carry a homozygous null mutation of the ephrin-A2 gene. Ephrin-A2 ^-/-^ mice were bred from heterozygous parents at the Biomedical Research Facility (The University of Western Australia) and backcrossed for >10 generations on a C57Bl/6J background. Randomised littermates were not used because the breeding colony was structured to produce ephrin-A2/A5 double knockout mice for other studies and no WT littermates were obtained. Mice were genotyped at weaning, as described previously ^21^. Mice were age matched, aged 8–10 weeks old (equivalent to young sexually mature adult in humans) when commencing the experiment. For the duration of the study, mice were kept in standard caging in a controlled environment (12/12 light/dark cycle; temperature 22°C±2°C, separated into cages with clear plastic walls (17 cm × 19 cm base, 16 cm high) based on sex and genotype (2–4 per cage)). Food restriction began two days prior to commencing training. This aimed to reduce mice to 90% of their free-feeding body weight. Mice were weighed daily and food intake adjusted using a daily-based controlled diet to reach and maintain target body weights and ensure animals remained healthy. Water was available *ad libitum* throughout the experiment. All procedures in this study were conducted in accordance with US NIH guidelines and approved by The University of Western Australia Animal Ethics Committee.

### Apparatus and procedure

Mice completed a visual discrimination task in two phases. Mice were initially rewarded for one stimulus (‘learning phase’). After mice learned the task (defined as 75% correct responses for three consecutive days), the rewarded stimulus was switched to the opposite, previously incorrect stimulus (‘reverse phase’). rTMS was applied (as described below) for 10 minutes daily immediately following the task during the reverse phase. We chose to stimulate after the task because we hypothesized that rTMS would enhance LTP-like processes, stabilizing new spines, and the associated synaptic connections, that had formed during learning. Because most mice in the study failed to learn the reverse task, we decided to focus on the relationship between rTMS and dendritic spine density, therefore, mice were terminally anesthetised with pentobarbitone (Lethabarb, Virbac Australia, 160 mg/kg, i.p.) 24 hours after 35 days of rTMS so that all mice received the same amount of stimulation.

### Visual discrimination task

The visual discrimination task was carried out using a Y-maze fitted into a 50 cm ^2^ box, with visual stimuli at each end of the Y maze arms (25 cm long; ^22^). Stimuli consisted of two 5 cm ^2^ laminated black and white striped cards at 0.37 cycles per degree oriented horizontally or vertically. Both genotypes are capable of distinguishing this spatial frequency ^26^. The position of the horizontal and vertical stimuli (left vs. right maze arm) was randomly altered across 30 trials with the constraint of equal number of trials in right and left arms. The 30 trial schedule changed each day, repeating every seven days. Random allocation determined which stimulus was rewarded in the learning phase, with the constraint that half the mice in each genotype received rewards for the horizontal and half for vertical. The rewarded stimulus was also counter-balanced across cage groups (i.e. mice housed together were rewarded for opposite stimuli) and sex. Inferential statistics confirmed no significant performance differences between sexes or stripe orientation first rewarded ( Data File). Mice were placed at the start of the Y-maze, and received a peanut butter reward immediately after approaching the correct stimulus. If mice did not approach a stimulus after 30 seconds, the trial was deemed a non-response (included in analyses as an incorrect response). Each mouse completed 30 trials per day, in a single session. The reverse phase commenced the day after mice reached criterion performance (75% correct for three consecutive days). In the reverse phase the opposite, previously incorrect stimulus was rewarded. All other aspects of the reverse task were identical to the initial learning phase.

### rTMS application

To deliver rTMS, we built a small coil created for mice (0.25 mm copper wire (Jaycar, Australia) 300 windings, 16 Ω, outer diameter 8 mm; ^23^). The coil was designed to ensure a similar coil-to brain ratio as is used for induction of focal electric fields in humans ^27^ and was driven by an electromagnetic pulse generator (Global Energy Medicine, Australia). Under these conditions, the coil delivered a magnetic field of 10 mT. This relatively low intensity was imposed by the constraints of the coil’s size but had the benefit of allowing us to evaluate the effects of stimulation without the confounding factors of stimulation-induced movement or the use of anaesthetic or restraint, with their associated changes to neuronal excitability and circulating stress hormones ^28^. Furthermore, low intensity magnetic fields are clinically relevant for two reasons. Firstly, in humans, fields in the millitesla range delivered to the brain induce analgesia ^29– 31^, and alleviate depression ^32^. Secondly, even though traditional rTMS is considered to be focal, magnetic fields of lower intensity are delivered outside of the focal area ^33^, raising the possibility that low intensity stimulation may be contributing to therapeutic effects by acting on interconnected brain regions.

We chose a complex pattern of stimulation that is based on biomimetic principles (described in detail ^23^ 59.9-ms trains of 20 pulses at 3 different frequencies as follows: 1 min warm-up at 6.71 Hz, 8 min treatment at 10.1 Hz, and 1 min cool down at 6.26 Hz) and has been shown to induce structural changes in mice ^23^. The pulse was monophasic with a 300µs rise time and 100µs fall time. A Hall device probe (Jaycar, Australia) inserted into different brain regions of a euthanized mouse estimated that the dorsal hippocampus received roughly 6 mT when the coil was held 1 mm above the mouse’s head, as described below. The surface temperature of the coil was measured after 10 min of stimulation and did not exceed 35°C.

As mice had completed the initial learning phase of the visual discrimination task before commencing rTMS they were accustomed to handling and remained relatively still without restraint. This allowed the stimulation coil to be held by the experimenter above the mouse’s head. We thus delivered reproducible rTMS in the awake animal (as for cat studies ^34, 35^). Unlike in cat studies, the coil was not in direct contact with the mouse head but was held as close as possible to the scalp (~1mm). The gap between the coil and the head does not attenuate the field because magnetic fields decrease with distance from the source but are not modified by air or biological tissue (e.g. skin/scalp ^36^). Unlike in the cat study, stereotaxic delivery was not attempted because the dimensions of the coil ensured that the field reached the entire dorsal hippocampus, which in the mouse, is relatively large in proportion to total brain size. Consistent with the low intensity of the magnetic field, mice did not display any head-eye or gross motor movements, nor altered behaviour in response to the stimulation. Sham stimulation involved the same procedure but with the stimulator switched off. This control was chosen as our coil did not produce any audible sound ^23^.

### Golgi staining

Terminally anesthetised mice were transcardially perfused with 4% paraformaldehyde (Sigma Aldrich; Montana USA); Right hemispheres of brains underwent a silver impregnation staining protocol, and a Golgi stain (according to manufacturer’s instructions: FD NeuroTechnologies, Maryland, USA), which allows the visualisation of morphology on a subset of neurons ^37^. Briefly, hemispheres were incubated in the dark in solutions A+B for 8 days with a change into fresh solution after the first 24 hours. Hemispheres were then incubated in solution C for 4 days with a change into fresh solution after 2 days. The impregnated hemispheres were then cryosectioned at 100 µm on a Leica Cryostat CM1900 at -19°C, mounted onto glass slides (Thermo Fisher Scientific, Australia) subbed with 0.5% gelatin (Sigma Aldrich, Montana USA). Sections were dried in the dark for 2–7 days, washed in distilled water and developed in solution D+E for 10 minutes. Sections were dehydrated in increasing concentrations of ethanol and mounted in Entilin (Merck, Darmastadt Germany).

### Imaging and analysis

Slides were analysed by a researcher blinded to stimulation condition and genotype. Sections were photographed by an Olympus DP70 digital camera at a 4× objective zoom, which encompassed the entire section. We analysed dendrites that could definitively be attributed to cells in the CA1 pyramidal and the molecular dentate layer of the hippocampus because dendritic spines on these cells have previously shown changes in dendritic spine density in response to various interventions ^38^. Dendrites were deemed suitable for analysis if they had a relatively flat orientation and were uniformly and strongly stained. Photographs of the dendritic arbour were taken throughout multiple planes ensuring the entire arbour was photographed in focus for later image analysis. Between 2 and 12 cells were analysed per animal and values averaged within animals for statistical analysis. Due to variability in Golgi staining, the number of dendrites counted varied between regions (CA1 pyramidal layer number of cells, Mean = 5; dentate molecular layer, Mean = 4.4).

The images for each dendritic arbour were combined into a single image, using the Image J plug-in "Stack Focuser" and dendrite length and number of dendritic spines counted using the Image J ("Cell counter" plug-in). The number of spines and the dendrite length data were then used to calculate a dendritic spine density value, defined as the number of spines per unit length (10 µm).

### Statistical analysis

We examined the effect of long-term rTMS on reverse learning performance in ephrin-A2 ^-/-^ and wildtype mice. Inferential statistics confirmed no pre-existing differences between groups in the learning phase, before commencing rTMS (data not shown). The first day of reverse phase training was excluded from analyses as rTMS commenced after this training session. Two mice reached the learning criterion in the reverse phase and were terminally anesthetised before 35 days (one wildtype and one ephrin-A2 ^-/-^, both received sham stimulation (negative/handling controls)) thus there was non-random reduction in sample size over time, precluding use of daily performance measures in statistical analyses. To overcome this problem, data were divided into three blocks for each subject, with one third of total days training included in each block, reflecting early, middle, and late stages of the reverse learning phase. Mean percentage correct was analysed by a two-way mixed ANOVA to assess differences between stimulation conditions (rTMS vs. sham) and between genotypes (ephrin-A2 ^-/-^ vs. wildtype) and changes over time (changes between early, middle and late blocks). As circuitry abnormalities and connections between measured hippocampal regions have not been characterised in ephrin-A2 ^-/-^ mice, it is unknown whether these measures should be considered independent for statistical analyses. Accordingly, a MANOVA was conducted to assess effects of stimulation condition (rTMS vs. sham) and genotype (wildtype vs. ephrin-A2 ^-/-^) on dendritic spine densities in both regions. Pillai’s Trace ( *V*) was used as the multivariate test statistic. Follow-up ANOVAs were conducted separately for each region, testing the same factors as used in the MANOVA. The F-test statistic ( *F*) and probability ( *p*) values are reported for each ANOVA. When the assumption of sphericity was violated degrees of freedom were adjusted with Greenhouse-Geisser correction. Data were analysed using SPSS statistics software (IBM Corporation, New York, USA, v.20).

Behavioural results (% accuracy) and average spine density for all mice Column 1: Mouse ID Column 2: Genotype (0=ephrin-A2-/-; 1=WT) Column 3: Stimulation condition (1=sham; 2=rTMS) Column 4: Gender (0=female; 1=male); Column 5: Stripe orientation (0=horizontal; 1=vertical) Column 6: Learning phase early (% accuracy) Column 7: Learning phase mid (% accuracy) Column 8: Learning phase late (% accuracy) Column 9: Reverse learning phase early (% accuracy) Column 10: Reverse learning phase mid (% accuracy) Column 11: Reverse learning phase late (% accuracy) Column 12: Dentate gyrus molecular layer, mean spine density per 10 micrometer Column 13: CA1 pyramidal layer, mean spine density per 10 micrometer

## Results

### rTMS had no significant effect on reverse learning performance

**Figure 1.**
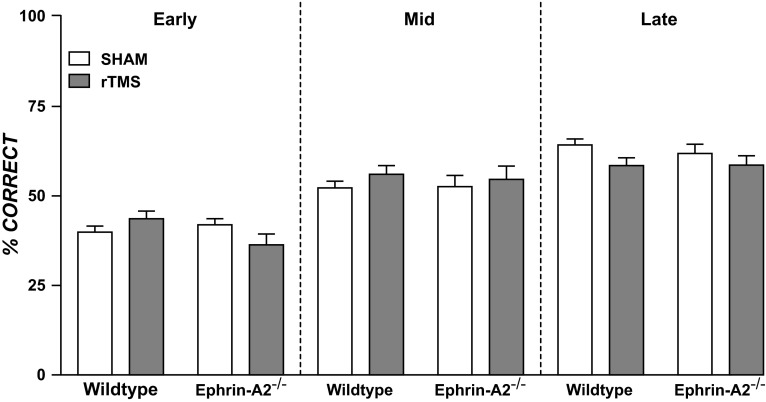
Mean percentage correct in a reverse-learning task for wildtype and ephrin-A2 ^-/-^ mice receiving daily rTMS. Early, middle (mid) and late blocks were delineated by the first, second and final third of total number of days training in the reverse-learning task. Within groups, scores increased significantly between blocks ( *p* <0.001), but there were no significant differences between rTMS and sham or between genotypes ( *p* values >0.05; ANOVA). Error bars = SEM.

As shown in Figure 1, groups had similar means in each block, with no significant difference between rTMS and sham, *F* (1, 16) = 0.28, *p* = 0.60, nor between genotypes, *F* (1, 16) = 0.86, *p* = 0.37, and no significant interactions across blocks (all *p* values >0.05). Within groups, the percentage correct increased significantly across blocks, indicating that mice adjusted to the rule reversal and successfully learned the task although this was not to the desired criterion ( *F* (1.43, 22.87) = 71.80, *p* < 0.001 (Greenhouse-Geisser corrected)).

### rTMS did not significantly affect hippocampal dendritic spine density

**Figure 2.**
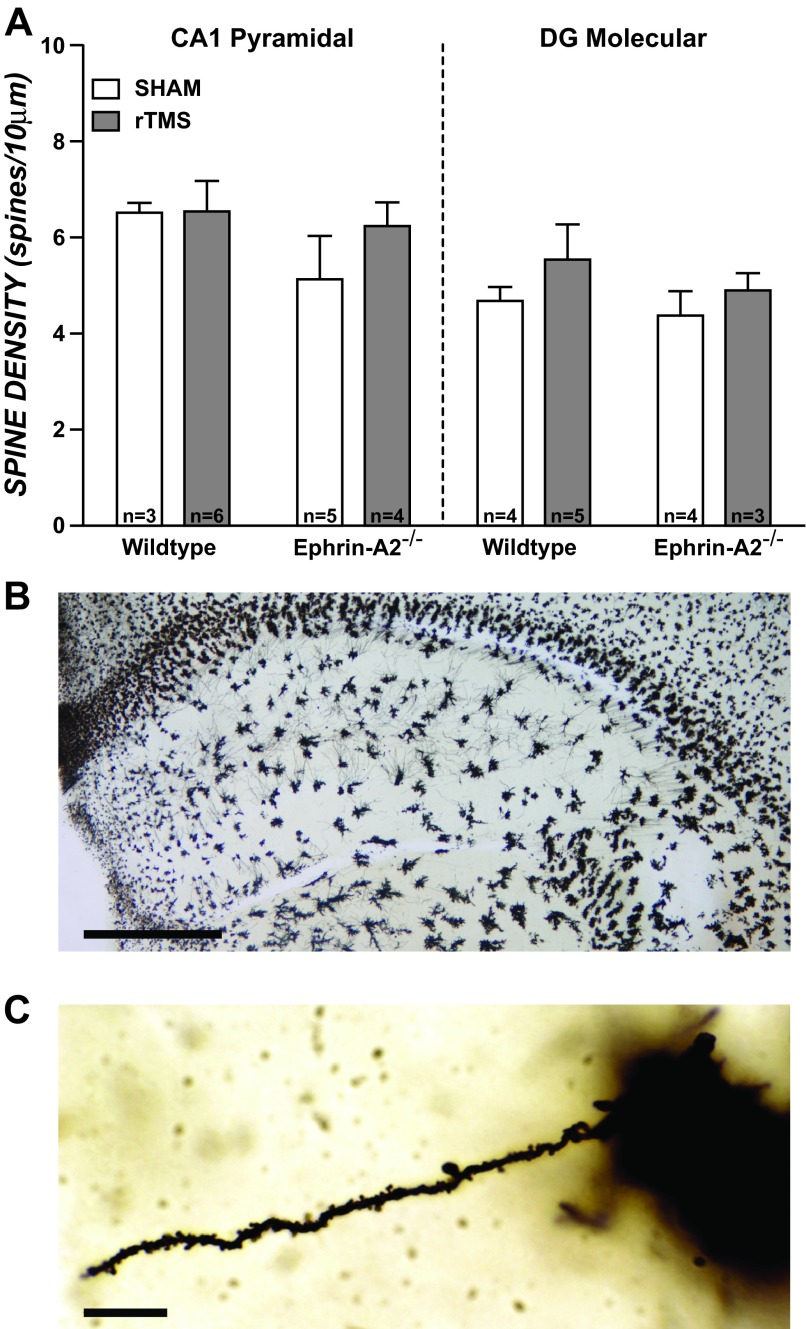
Assessment of rTMS effects on dendritic spine density in wildtype and ephrin-A2 ^-/-^ knockout mice. ( **A**) Mean dendritic spine density (number of spines per 10 μm) for hippocampal regions: CA1 pyramidal layer and dentate gyrus molecular layer (DG Molecular). There were no significant effects of rTMS nor genotype on spine densities in either region ( *p* values >0.05; ANOVA). Error bars = SEM. ( **B**) Right hemisphere Golgi stained section of dorsal hippocampus representative of those used in analyses. Scale bar represents 500 μm. ( **C**) Dendrite representative of those selected for analysis, with spines visible. Scale bar represents 10 μm.

Spine density (spine number/10 µm) measures were obtained for each dendrite and averaged within mice. Figure 2 presents mean dendritic spine density in CA1 pyramidal layer and dentate molecular layer for ephrin-A2 ^-/-^ and wildtype mice, contrasting rTMS to sham. There was a slight (non-significant) trend towards rTMS increasing spine density in ephrin-A2 ^-/-^ mice in both regions. In wildtypes rTMS appeared to have no effect on spine densities in CA1 pyramidal cells, as means were almost identical. However, wildtypes showed a similar (non-significant) trend to ephrin-A2 ^-/-^ mice in the dentate molecular layer.

MANOVA, using Pillai’s trace showed there was no significant effect of stimulation condition, *V* = 0.13, *F* (2, 9) = 0.67, *p* = 0.54, nor genotype, *V* = 0.08, *F* (2, 9) = 0.39, *p* = 0.69, on spine densities. Follow up two-way ANOVAs were also performed separately for each region, with no significant differences between stimulation conditions or between genotypes in either region, all *p* values were >0.05 (CA1 pyramidal layer: stimulation condition, *F* (1, 10) = 0.03, *p* = 0.86; genotype, *F* (1, 10) = 0.86, *p* = 0.38. Dentate molecular layer: stimulation condition, *F* (1, 10) = 1.03, *p* = 0.34; genotype, *F* (1, 10) = 0.42, *p* = 0.53).

## Discussion

We investigated the effects of long-term daily rTMS on learning and hippocampal dendritic spine density using ephrin-A2 ^-/-^ mice and wildtype controls. We show that rTMS had no significant effect on learning and no significant effect on hippocampal dendritic spine densities. Although ephrin-A2 ^-/-^ mice have abnormal brain circuitry and associated abnormal behaviours, in the present study, the previously reported learning deficit ^18^ was not observed due to low levels of food deprivation ^22^. Although it is difficult to draw conclusions from the null results presented here, the absence of observed behavioural and structural change is consistent with previously reported rTMS specificity for abnormal systems ^8, 23^. The lack of adverse effects in our long-term study suggests that up to 5 weeks of daily sessions of low intensity pulsed magnetic field stimulation at the parameters used in this study appears safe to use in healthy participants.

### Long-term rTMS does not adversely affect learning in normal subjects

We originally hypothesized that rTMS would rescue the learning strategy deficit in ephrin-A2 ^-/-^ mice ^18^ with minimal or no effect on wildtype mice. However, both genotypes failed to demonstrate the strategy deficit, due to insufficient food restriction ^22^. Our results nonetheless indicate that rTMS does not adversely affect performance, but nor does it improve motivation or accelerate learning when deficits are absent. This is consistent with previous reports that long-term rTMS effects are specific to abnormal brain circuitry: two weeks of rTMS improved visual tracking, visual electrophysiological function and topographical accuracy in a different strain of mice (ephrin-A2A5 ^-/-^ double knockouts) with abnormal circuitry but produced no lasting effects in wildtype mice ^23^. Human studies also support specificity of rTMS for abnormal brain circuits, as a meta-analysis of rTMS effects on cognitive performance found patients tend to improve more than healthy participants ^8^. Although some human studies using healthy participants show a single-session of rTMS enhances cognitive task performance, such as analogous reasoning ^39^ and reaction time ^7^, results are mixed, with other studies showing no effect of rTMS on knowledge acquisition ^40^ or accuracy in a go/no-go task ^41^. Furthermore, there is a lack of studies assessing cognitive effects of long-term rTMS in patients together with healthy controls, which presents a large gap in knowledge ^8^. Because of the lack of understanding of fundamental interactions between rTMS and behaviour, it would be of great interest to perform an exhaustive battery of behavioural tests in healthy wildtype mice (and eventually in animal models of disease) in conjunction with various rTMS protocols. Subsequent anatomical and physiological analyses could then be carried out to elucidate the neural mechanisms of rTMS and gain insight into the treatment of human disease.

### Long-term rTMS and hippocampal spine density

Each mouse received a controlled amount of daily rTMS, allowing us to investigate how long-term rTMS combined with daily training influences spine density. Importantly, we found similar spine densities in sham wildtype and ephrin-A2 ^-/-^ mice, suggesting that spine density is not solely dependent on ephrin-A2, in agreement with the literature ^19– 21^. As such, the null effect of rTMS on dendritic spine density may be attributed to the absence of both a specific spine and learning deficit in both wildtype and ephrin-A2 ^-/-^ mice. It will be important to examine other brain regions to determine whether the selectivity of rTMS for normal and abnormal brain circuits is also observed. Our result that spine density was not significantly altered is in agreement with a previous study, showing no change in spine density in CA1 pyramidal neurons following a single rTMS stimulation ^2^. However, it is surprising that dendritic spine density remains unaffected after long-term stimulation, given our previous results using the same stimulation parameters, demonstrating structural reorganisation in abnormal axon terminals following multiple stimulations, but not a single rTMS session ^23^. As neither long-term nor short-term rTMS results in dendritic spine density changes, these negative results may suggest different susceptibility of axons and dendrites to rTMS. The functional characteristics of these neuronal compartments require different expression of ion channels and growth factor receptors ^42^, which could provide a molecular basis for differential rTMS effects on excitability and spatially localised structural and functional change.

Alternatively, the timing of rTMS delivery relative to the behavioural task may have influenced the outcome of our experiments. Here we stimulated after the task, however rTMS might have been more effective if delivered before. Because a single session of rTMS increases the size of dendritic spines and may activate silent synapses ^2^, this may “prime” the brain for learning. With such pre-treatment, an effect of rTMS might even have been detected in improved performances on a day to day basis.

An alternative interpretation of our null finding is that our rTMS treatment changes spine dynamics without affecting their final density, a result that would not be possible to detect in our fixed post-mortem tissue. Hence, these results highlight the limitations in Golgi staining of fixed tissue, a technique still commonly used in examining dendritic spine density. Sensory manipulation (either enrichment or withdrawal) strongly alters spine dynamics *in vivo* in various areas of the cortex of adult mice ^43^. As a rule, established spines are pruned during the initial experience of the new stimulus, while new ones are established, which may result in some studies of fixed tissue showing no net change in spine density ^44, 45^. Consistent with the change in spine dynamics initiated by enrichment, a recent imaging study in the hippocampus identified two phases in spine dynamics following repeated induction of LTP. Initially both generation and retraction of spines increased, followed by a cessation of spine retraction ^46^. This is consistent with post-mortem studies showing an initial period of apparent spine stability, followed by a detectable increase in density. The possibility that rTMS changes spine dynamics, as opposed to density, is further supported by an increase in the size of small spines following a single stimulation, which the authors suggested may indicate the activation of silent synapses by membrane recruitment of AMPA receptors, precluding the need for de novo synapse generation ^2^. Future live imaging studies of spine dynamics in animals that have received single or multiple rTMS stimulation, potentially in combination with learning tasks will provide much needed insight into the mechanisms underpinning the plastic changes elicited by rTMS in humans.

Importantly, we are conscious of the limitations of our rodent scaled rTMS delivery device which may have contributed to the lack of effect observed here. Although our coil had a relevant coil to brain ratio for mice, because of its small size, the intensity of the magnetic field did not reach the magnitude commonly used in humans (6mT compared to 1-2T), raising concern that our stimulation paradigm is not comparable to human rTMS. However, this raises a more general issue because similar criticism applies to studies that employ larger coils ^1– 3^: although these deliver the same fields used in humans, the focal nature of the stimulation is lost. Additional effort in designing appropriate small animal rTMS coils is urgently needed to improve the construct validity of animal rTMS research.
