# Supplementary material for: Long term delivery of pulsed magnetic fields does not alter visual discrimination learning or dendritic spine density in the mouse CA1 pyramidal or dentate gyrus neurons
Source: F1000Res. 2013 Dec 4;2:180. Originally published 2013 Sep 9. [Version 2] doi: 10.12688/f1000research.2-180.v2 (PMC3938248; doi:10.12688/f1000research.2-180.v2)
Supplement: Peer review history

# Peer review: Referee response for version 2

**DOI:** 10.5256/f1000research.3188.r4012

**Published:** 2014-03-10

**Authors:** Anthony Hannan

**Affiliations:** 1 Behavioural Neuroscience, The Florey Institute of Neuroscience and Mental Health, Victoria, Australia

I have read this submission. I believe that I have an appropriate level of expertise to confirm that it is of an acceptable scientific standard.

# Peer review: Referee response for version 1

**DOI:** 10.5256/f1000research.2167.r2135

**Published:** 2013-10-21

**Authors:** Anthony Hannan

**Affiliations:** 1 Behavioural Neuroscience, The Florey Institute of Neuroscience and Mental Health, Victoria, Australia

This is a well written paper describing the effects (or lack thereof) of rTMS over 5 weeks in wild-type and ephrin-a2 knockout (KO) mice. The data are clearly presented in a usable format and are discussed appropriately. The initial referee Antoni Valero-Cabre, who is an expert in TMS and neuro-stimulation, has already extensively covered the key points, both positive and negative. I am therefore only going to make a few additional comments.

The title and abstract should ideally reflect the findings of the study more precisely, for example:

‘Long-term delivery of pulsed magnetic fields does not alter visual discrimination learning or dendritic spine density in mouse CA1 pyramidal or dentate gyrus neurons’.

The nature of such negative findings means that the authors cannot rule out potential significant effects of this rTMS protocol on other learning tasks or other classes of neurons/dendrites in the mouse hippocampus. Furthermore, the abstract should mention that they were adult mice and the background strain used was C57Bl/6J, as it is possible that different aged mice and/or a different genetic strain of mice might respond differently to the same rTMS protocol.

One concern regarding the methods and design is that the authors appear to have used a separate wild-type mouse colony and compared them to ephrin-a2 KO mice inbred via a backcrossed colony. I strongly feel that all such experiments should always involve wild-type littermates randomised from age-matched litters to control for both genetic (e.g. sub-strain and genetic drift) and epigenetic differences between different colonies. This is particularly important for behavioural experiments where even subtle genetic and epigenetic differences can often have significant impacts. The authors report no significant gene effects, and it is therefore not a major confound in this case, however if randomised littermates were not used (as the methods imply) this should be noted.

The discussion and conclusions are balanced. However, it might be worth noting that an extensive battery of behavioural tests (for example including sensory, cognitive, affective and motor protocols) on wild-type mice would be worth pursuing to assess whether this rTMS protocol (or others with different spatial and temporal specificities) has any effects on brain function analogous to human studies. If any positive results were found, then cellular, physiological and molecular follow-up studies could be targeted towards understand specific cognitive/behavioural effects of rTMS (thus nicely complementing human studies).

If such comprehensive TMS animal studies are done but still show inconsistencies with matching human TMS studies then one possibility is that the animal TMS needs to better match the exact spatial, temporal and biophysical aspects of human TMS. For example, the human brain is much larger, the skull much thicker and the spatial extent and physical effects of a given TMS coil are no doubt difficult to match. The need to further improve such ‘construct validity’ for animal TMS may also be worthy of further discussion.

I have read this submission. I believe that I have an appropriate level of expertise to confirm that it is of an acceptable scientific standard.

# Peer review: Referee response for version 1

**DOI:** 10.5256/f1000research.2167.r1747

**Published:** 2013-09-26

**Authors:** Antoni Valero-Cabre

**Affiliations:** 1 School of Medicine, Anatomy and Neurobiology, Boston University, Boston, MA, USA

This is an outstandingly planned and well-executed study that shows that the rodent brain, after a daily 10 minute exposure to low field pulsed magnetic stimulation for 5 weeks (i.e. 35 sessions) exhibits null effects on learning behavior and hippocampus dendritic spine density in both Wild Type and Ephrine A2-/- mice. The effort of the authors deserves a lot of credit, as studies like this one are extremely hard, long and laborious to perform and the possibility of significant results always uncertain. In spite of the current null results, this kind of work is greatly needed in the field of non-invasive brain stimulation in order to understand the cellular underpinnings of these techniques and assess the safeness and real therapeutic potential of stimulation technologies in clinical neurorehabilitation. Overall, the neurostimulation community should strongly thank the authors for investing their know-how, time and effort to carry over this type of animal research and we all should encourage them to pursue their efforts in the future.

**GENERAL POINTS**

The only limitation of the current manuscript is related to the difficulties in interpreting null outcomes; in this case a triple negative result. Not only did low field pulsed magnetic stimulation prove unable to modify visual learning behavior or hippocampus spine density in these two population of mice but neither of the two showed (as should have been at least expected) significant differences in any of these two measures. The authors do an excellent job going over the different possibilities that could explain this null pattern of outcomes, even if they do not have much basis to rule out any of the hypotheses in particular. Among other important issues, the study emphasizes once more the importance of task titration. Task titration is strongly related to animal motivation (and thus intake restriction) and is a key variable and therefore needs to be manipulated carefully to make sure that the potentially effective impact of magnetic pulses can be demonstrated behaviorally. With regards to the question however of whether or not increasing food restriction could have rendered, as the authors suggest, behavioral learning tests more sensitive, the answer remains unknown. It could also be that highly motivated animals would rapidly show ceiling learning effects and render the task less sensitive to modulation. To this reviewer, the performance of both groups of animals in the inversion phase of the task seems to show enough room for behavioral improvement, even after 5 weeks of stimulation; thus it is tempting to also speculate that such a complex task for a rodent (which relies on several cognitive processes such as visuo-spatial attention, visual perception, visual, spatial and declarative memory, associative rewarded learning, rule understanding and switching and decision making) may not have been well suited to the functions of the brain regions aimed at in this study and/or that their impact could have rapidly compensated for by other systems or regions.

Adding to the authors’ arguments with regards to the lack of spine density effects, one could also argue that a direct impact of the stimulation (and not an indirect effect of the stimulation on learning behavior driving subsequent effects on memory related regions) on such measures could and should have occurred primarily in the cortical regions overlying the hippocampus (which have not been analysed in this study) rather than in the more deeply located hippocampus CA1 and DG neurons. Of course, if the targeted region theoretically related to the behavior that is being measured is not at least directly or indirectly impacted and modified in activity by the pulses, then no behavioral differences between sham and real rTMS patterns should be expected in either population of mice. All these explanations are directly or indirectly mentioned in the discussion and when not explicitly mentioned in the text, they emerge logically from the results. However any effort to nuance and expand such justifications even further would be welcome. 

To this reviewer, the only possibility (that would also be compatible with the negative outcomes of this study) that is not explicitly mentioned in the discussion is that the delivered low field pulsed magnetic patterns (even if estimated by means of a Hall device in postmortem brains as reaching intensities of ~6 mT in the hippocampus) were not effective at all. Several reasons that could account for that eventuality are; that the field was too weak to penetrate deep enough with a minimal intensity, imprecise widely distributed targeting or a field strongly attenuated by the space left, as indicated in the manuscript, between the stimulating coil and the top of the head. This reviewer cannot demonstrate that this possibility is more likely than any of the others already mentioned in the manuscript. Nonetheless, in the absence of any positive sign of a stimulatory impact, this possibility cannot be ruled out and should probably be briefly discussed. Additionally, it should be clear throughout the manuscript, that the ability of the current experiment to highlight the innocuity of 5 weeks of low field pulsed magnetic brain stimulation needs to be interpreted carefully and associated with the field strengths delivered by the modified e-cell device by Global Energy Medicine, which is far from the normal field strength for TMS/rTMS devices operated in humans for either research or therapeutic purposes. 

**MINOR COMMENTS AND SUGGESTIONS**

1. *Introduction, Page 1, Col 1, Par 1, Line 6. *Could the authors re-evaluate the adequacy of the term “metaplasticity” with regards to TMS-induced LTP effects, as to this reviewer, the main effect of high frequency stimulation are LTP or LTP-like effects. Metaplasticity is a consequence of forcing homeostatic plasticity beyond a particular boundary, which may result in paradoxical modulatory effects.

1. *Introduction, Page 1,  Col 1, Par 2, Lines 7-9*. With regards to this issue, in this particular study were the authors hypothesizing a direct impact of low field pulsed magnetic stimulation on specific cortical locations translated through connectivity into a declarative and spatial memory region such as the hippocampus, or a cortical effect of stimulation leading to subsequent changes in behavior that could leave a distinctive memory trace in the hippocampus?

1. *Introduction, Page 1, Col 1, Par 3*. The paper by *May et al.* *2007, Cereb. Cortex,* in which the effect of 5 straight days of rTMS on humans assessed with MRI methods and regional size increases (hypothetically attributed to increases in spine density) could be relevant for this introduction.

1. *Methods, Page 1, Col 2, Par 1, Lines 7-8.* Could the authors provide an idea of how far in their development (if possible in age compared to humans) are 8-10 week old mice? It seems that in a prior study by the same authors that showed significant results after 14 days of low field rTMS on the visual cortex by *Rodger et al.* ***2012, FASEB J,* the mice were slightly younger (6-8 weeks). Is this period crucial in terms of postnatal development for mice? Could a less plastically sensitive brain of 8-10 week old mice vs. 6-8 week old mice explain the current null results? Please comment briefly in the manuscript.

1. *Methods, Page 2, Col 2, Par 2, Lines 6-7.* Why were mice tested before the low field pulsed magnetic stimulation session and not also immediately thereafter, when the impact of the stimulation should have been stronger? Although I understand the authors sought a long-term effect, such a measure could have proven useful to reveal at least an immediate day-to-day impact of stimulation and become a proof of their efficacy?  Given the order of events, one could be tempted to speculate the possibility of non-synergistic interaction between the lasting effects of task practice and immediately subsequent low field rTMS stimulation, cancelling the modulatory effects.

1. *Methods, Page 3, Col 1, Par 2, Lines 7-8*. As this is not a standard rTMS stimulation device, some additional information on the stimulation source should be given to be able to compare the efficiency of low field pulsed magnetic stimulation with current human rTMS equipment. More specifically, what is the shape of the stimulating pulse (monophasic, biphasic?) and what is the rise time of the field? The pattern of stimulation used seems essentially an “excitatory” 10 Hz rTMS pattern, preceded and followed by short instances of ~6 Hz stimulation. Was this high frequency employed as a way to induce LTP phenomena in the hippocampus or as a tool to enhance cortical excitability and facilitate learning behavior leading then to functional and anatomical modulations of the hippocampus? Please comment briefly.

1. *Methods, Page 3, Col 1, Par 2, Lines 7-8. *Navigation of the TMS coil of course could have been improved given the high-focality of the device used by the authors and the fact that any small head movements in a small rodent brain could easily lead to a completely different area of stimulation. It is true that prior studies in awake felines by our own group (see *Valero-Cabré et al. 2006, Exp Brain*, *Valero-Cabre A et al.* **2008,* Eur J Neurosci.*) have used a similar manual procedure, but it is also the case that the coils and brains in those studies were larger and precise location on a specific region of the posterior parietal cortex was guided day to day by stereotaxic based measures and references. In many instances, we additionally benefited from parietal bone transparency during surgery, and identified in each animal the sulci/gyral pattern and labelled the exact area of interest (see *Valero-Cabré et al. 2005 Exp Brain Res, Valero-Cabré et al.2007 Exp Brain Res*). Furthermore, in groups that were to be followed longitudinally, we placed a dental acrylic plug, that could be palpated through the skin of the scalp (see *Afifi et al. 2012 Eur J Neurosci*) which was then used as consistent localization marker. The correct positioning of those plugs with regards to the stimulated areas was often verified pre-treatment by anatomical MRI methods and also in post-mortem brain dissections at the end of the follow up. In the current study, given the elongated shape of the hippocampus in rodents, which part of the rodent cortex or which stereotaxic level was aimed at during the stimulation? Also, in contrast with the quoted feline study, the authors indicate here that the coil was not in direct contact with the scalp. As air is a strong isolator, could that have attenuated the strength of such a low intensity field even further? Were Hall probe field measures in the hippocampus performed with the stimulating cell also separated from the scalp?

1. *Discussion, Page 5, Col 1, Par 1, Lines 4-6.* The statement “ *The absence of observed behavioral and structural change is consistent with previously reported rTMS specificity for abnormal systems*” needs to be expanded upon as the Ephrin-A2-/- mice have indeed abnormal systems. Maybe a short comment on how to reconcile the current findings with those reported in *Rodger et al.* ***2012, FASEB J*, would be important as in that study by the same authors similar stimulation patterns were able to correct cortico-collicular connectivity only in Ephrin-A2-/- and not in Wild type mice, supporting the above-mentioned statement.

1. *Discussion, Page 5, Col 1, Par 1, Lines 6-8*. I would strongly advise the authors to alter the contents of the sentence “ *Furthermore, the lack of adverse effects in our long term study contributes evidence that rTMS is safe to use in healthy control participants*”. Could they rephrase to something like “Furthermore, the lack of adverse effects in our long term study suggests that up to 5 weeks of daily sessions (35 session) of low field pulsed magnetic stimulation at the parameters used in this study appears safe to use in healthy participants”. This is important as the intensity levels employed are very different of those employed in human rTMS, which are much higher, and this information could have some public health implications for future use of rTMS in humans.

1. *Discussion, Page 5, Col 2, Par 1, Lines 19-21*. Please check the accuracy of the sentence “ *To our knowledge, there have been no studies assessing cognitive effects of long-term rTMS in patients and healthy controls, which presents a large gap in knowledge*”

1. *Discussion, Page 5, Col 2, Par 1, Lines 21-22*.  As indicated above (comment 10), would the authors agree to rewrite the sentence “ *Although more research is needed, our results support that rTMS is safe to use in healthy control participants…*” and revise the second part of it  “ *… invaluable in assessment of rTMS effects clinically*” which to this reviewer is confusing?

1. *Discussion, Page 5, Col 2, Par 2, Lines 5-8.* This sentence can be a bit surprising to readers, why did the authors chose a knock out for Ephrine A2 in a study that aimed to analyze spine density in the hippocampus if as they affirm in this paragraph, this molecule is not involved in spine dynamics? Maybe the rational for that choice needs to be explained more carefully.

I have read this submission. I believe that I have an appropriate level of expertise to confirm that it is of an acceptable scientific standard.
